# Influence of the Morphology of Core-Shell Supports on the Immobilization of Lipase B from *Candida antarctica*

**DOI:** 10.3390/molecules190812509

**Published:** 2014-08-18

**Authors:** Martina C. C. Pinto, Denise M. G. Freire, José Carlos Pinto

**Affiliations:** 1 Programa de Engenharia Química, COPPE, Universidade Federal do Rio de Janeiro, Rio de Janeiro, Caixa 68502, Brazil; E-Mail: mpinto@peq.coppe.ufrj.br; 2 Departamento de Bioquímica, Instituto de Química, Universidade Federal do Rio de Janeiro, Rio de Janeiro, Caixa 68502, Brazil; E-Mail: freire@iq.ufrj.br

**Keywords:** core-shell particles, polystyrene, immobilization, lipase B from *Candida antarctica*

## Abstract

Core-shell polymer particles with different properties were produced through combined suspension-emulsion polymerizations and employed as supports for immobilization of lipase B from *Candida antarctica*. In order to evaluate how the morphology of the particles affects the immobilization parameters, empirical models were developed to describe the performance of the biocatalysts as a function of the specific area, volume of pores and average pore diameter of the supports. It was observed that the average pore sizes did not affect the enzymatic activities in the analyzed range of pore sizes. It was also observed that the increase of the specific area (and of the volume of pores) led to higher enzyme loadings, also leading to an increase in the esterification activity, as expected. However, when the specific area (and volume of pores) increased, the hydrolytic activity and the retention of hydrolytic activity of the biocatalysts decreased, indicating the existence of diffusional limitations for some hydrolytic reactions, probably because of the high reaction rates.

## 1. Introduction

Among enzymes, lipases have received considerable attention both in the scientific literature and in the commercial market [1]. Lipases represent about 5% of the world enzyme market and, because of their wide-ranging applications, this market is expected to grow in the coming years [2]. Lipases can catalyze different reactions, including hydrolysis, esterification and transesterification reactions, with high selectivity and specificity [2,3,4,5]. For this reason, lipases find widespread use in many different areas, including applications in the food, pharmaceutical, cosmetics and biosensors fields [1,6]. Lipases can also be employed for the treatment of effluents [7,8] and the production of biodiesel [9,10,11].

Nevertheless, soluble enzymes cannot be recovered easily at the end of the reaction process, which limits the development of continuous operations and increases the operation costs, as enzymes with the desired degree of purity are usually very expensive. In order to minimize these problems, it is normally recommended that enzymes be employed in the immobilized form [12], facilitating the recovery of the biocatalysts at the end of the process, enabling the conduction of continuous process operations and allowing for enzyme reuse [12,13,14,15]. Additionally, some enzymatic properties can also be enhanced by the immobilization process, such as activity, selectivity, specificity and stability [12,14,15]. Improvement of enzyme performance can be related to modification of the enzyme structure (due to chemical and physical interaction with the support), generation of a more favorable reaction environment in the surroundings of the enzymes (due to interaction of the support with the reactants and solvents), existence of diffusional limitations (modifying concentration gradients along the pores), among others [16,17,18,19,20,21].

Enzyme immobilization can be performed through chemical reactions or chemical and physical interactions between the enzymes and the supports [11,12,15]. However, it is important to note that, depending on the particular method employed for enzyme immobilization, the produced biocatalyst may present different properties. The most common method employed for lipase immobilization is the adsorption of the enzyme on the surface of a solid support [2,5,11].

It is interesting to observe that most lipases present a lid that covers the active center of the enzyme molecule. However, in contact with hydrophobic surfaces these enzymes show an open form, exposing the hydrophobic pocket of the enzyme and enhancing the enzymatic activity [5]. One of the most used lipases is lipase B from *Candida antarctica* (CAL-B) [2,22,23]. Although the structure of this enzyme presents a very small lid, that does not completely block its active center [2,24], it is able to absorb onto hydrophobic carriers [2,25]. The main properties of CAL-B have been discussed by many authors and can be found elsewhere [2,22,23,24,25]. However, it is important to notice that CAL-B is a globular protein constituted by 317 aminoacids and presenting a molecular weight of 33 kDa and isoelectric point of 6.0.

There are many supports used for lipase immobilization, including natural polymers, such as chitin, chitosan, gelatin, dextran and cellulose, and synthetic polymers, such as polyacrylamide, poly(vinyl alcohol), polystyrene, and others [26,27]. As the properties of the support can affect the activity of the biocatalyst, many studies are being conducted to allow for development of new synthetic polymer supports, since most polymers can be synthesized easily and at low cost.

In this context, core-shell polymer particles produced through combined suspension/emulsion polymerizations can be potentially employed as supports for cell and enzyme immobilizations [28]. These supports can exhibit very porous morphology and adsorb high amounts of proteins [2,28,29,30]. Moreover, the combined suspension/emulsion polymerization process enables the easy modification of the chemical and physical properties of the support surfaces, allowing for adjustment of the support properties in order to improve the interaction of the particularly analyzed enzyme with the support, leading to tailor-made supports for specific enzymes [2,29]. Furthermore, particles can be synthesized in a single process step, not requiring necessarily the implementation of additional process steps for functionalization of the final polymer particles [29].

Figueiredo *et al.*, employed porous polystyrene core-shell particles produced through combined suspension/emulsion polymerization for the adsorption of lysozyme, aiming at separating this protein from the protein medium [30]. It was observed that large amounts of protein could be recovered with the help of the core-shell particles with low costs. More recently, Cunha *et al.*, employed similar core-shell polymer particles for immobilization of commercial lipase B from *Candida antarctica* (CAL-B), obtaining performances for esterification and hydrolysis that were better than reported for other commercial supports [2].

More recently, Besteti *et al.*, prepared different enzymatic CAL-B catalysts and observed that more active biocatalysts (based on hydrolytic activity) could be obtained with core-shell polymer supports containing polystyrene in the core and poly (methyl methacrylate) in the shell [31]. According to the authors, the presence of polar monomers in the shell can improve the efficiency and yield of immobilization and retention of activity of the immobilized CAL-B, as a consequence of the less intense interaction established between the enzyme and the support, which prevents distortions of the tertiary structure of the enzyme. Despite that fact, Besteti *et al.* did not analyze the effect of polymerization conditions and final polymer support morphology on the performance of the final biocatalysts [31]. For this reason, it becomes necessary to produce supports with different morphological properties (with distinct specific areas, pore diameters and volume of pores) and to evaluate how the support characteristics affect the immobilization parameters. This can allow for optimization of CAL-B performance in many applications, such as those related to the kinetic resolution of myo-inositol derivatives [32,33,34,35,36].

Based on the previous paragraphs, the main objective of the present work was to characterize how the morphology of core-shell polystyrene particles produced through combined suspension/emulsion polymerizations (specific area, volume of pores and average pore diameter) affects the immobilization parameters of CAL-B (such as immobilization yield and retention of enzymatic activity in hydrolytic and esterification reactions). In order to do that, core-shell particles with different properties were initially prepared through manipulation of some operation parameters of the combined suspension/emulsion polymerization process. Afterwards, the immobilization of CAL-B on the previously prepared particles was performed through physical adsorption. Finally, the immobilization parameters were analyzed quantitatively with help of empirical models and standard statistical analysis. Polystyrene was used because this polymer presents high hydrophobicity and good mechanical strength, while CAL-B is one of the most used lipases [2,22,23]. 

## 2. Results and Discussion

### 2.1. CAL-B Immobilization

**Figure 1:**
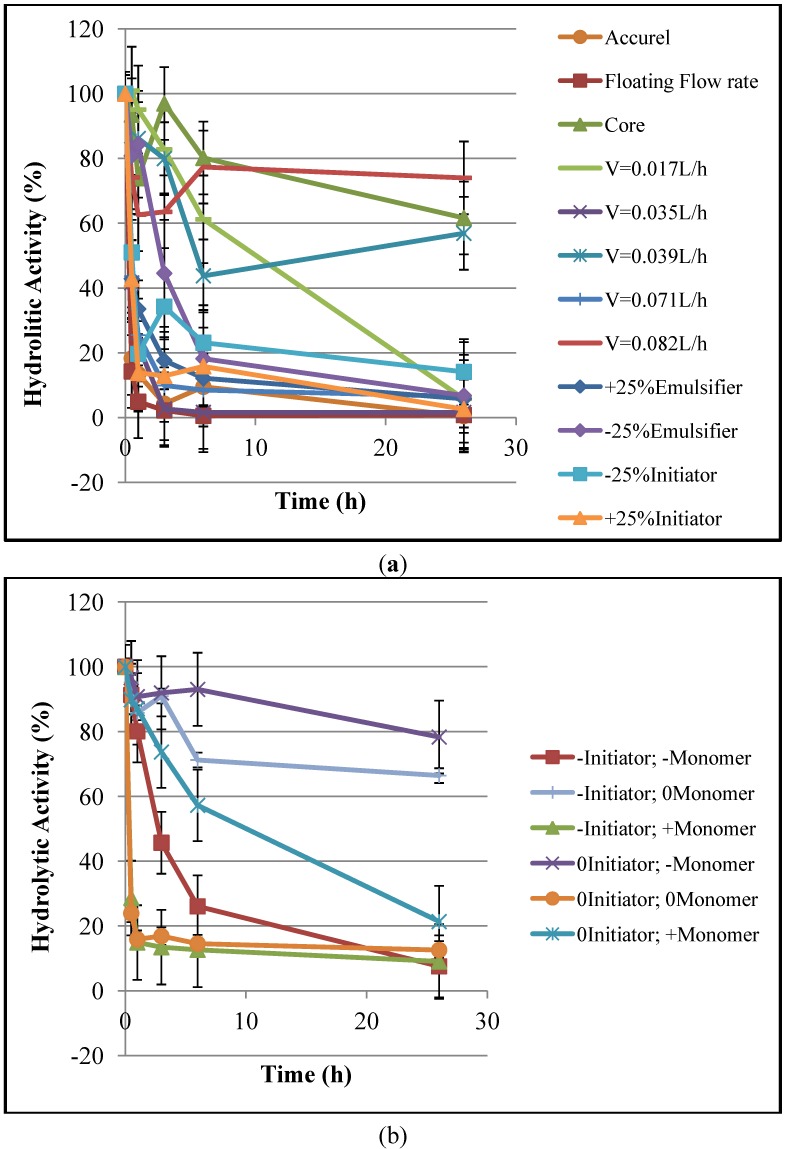
Kinetics of the enzyme immobilization based on the hydrolytic activity: (**a**) first group of supports and (**b**) second group of supports.

**Figure 2:**
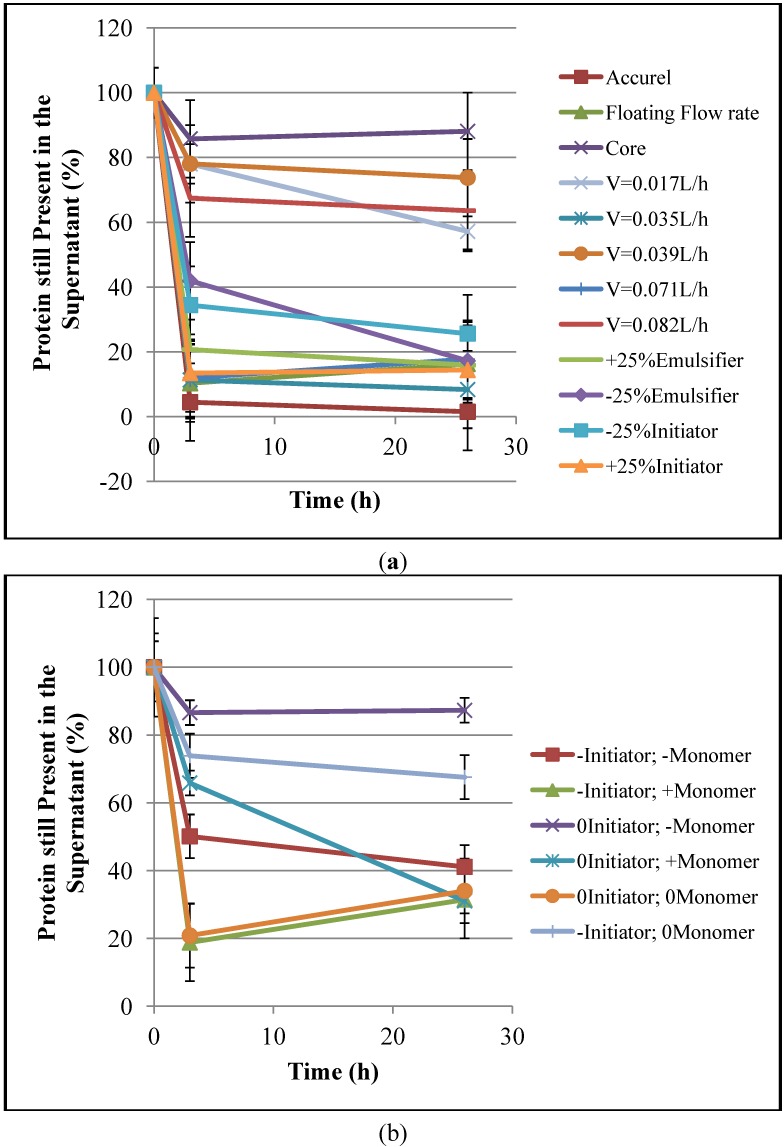
Kinetics of the enzyme immobilization based on the protein concentration: (**a**) first group of supports and (**b**) second group of supports.

Figure 1 illustrates the enzyme immobilization kinetics, based on the hydrolytic activity, for different supports. It is possible to observe that most supports are able to absorb more than 80% of the enzyme activity of the initial solution under the analyzed immobilization conditions. It is particularly important to mention that the supports that adsorbed the smallest amounts of enzyme also presented the lowest specific areas. It is also important to observe that, although the core particles exhibited very low specific areas, significant amounts of enzyme were absorbed by core particles, probably because of the interaction forces that exist between the lipases and the hydrophobic surfaces of the supports [5]. Figure 2 shows the evolution of protein concentrations in the aqueous solution during the enzyme immobilization experiments, confirming the results of Figure 1 and showing that most supports could absorb more than 60% of the initial amount of protein. This supports the idea that the observed decrease of hydrolytic activity was caused by the increase of the amount of protein adsorbed onto the supports surfaces during the immobilization process.

### 2.2. Influence of the Morphology of the Supports on the Immobilization Parameters

The immobilization parameters obtained for each immobilization experiment are listed in Table 1. The analysis of these parameters is essential for the understanding of the effect of the morphology of the supports on the performances of the produced biocatalysts. As the immobilization experiments were performed at different moments, the initial hydrolytic enzymes activities are not equal, but always in the vicinities of 100 U.

**Table 1:** Immobilization parameters obtained for the different immobilization supports.

Supports	Ue (U/gsupport)	Ahid (U/gbio)	Aester (U/gbio)	Utheo (U/gsupport)	Η (%)	Ra (%)	Protein Concentration on the Support (mg/gsupport)
Accurel	91.8	0.7 ± 0.7	616 ± 166	91.4	99.5	0.7	4.5
Carrier 1	127.0	2.3 ± 0.6	588 ± 307	126.0	99.2	1.8	5.2
Carrier 2	84.4	5.7 ± 2.4	133 ± 111	32.4	38.4	17.6	0.5
Carrier 3	116.8	4.2 ± 2.5	311 ± 80	115.0	98.4	3.6	5.3
Carrier 4	80.7	2.2 ± 1.1	56 ± 21	34.8	43.1	6.3	1.0
Carrier 5	91.6	0.9 ± 0.9	193 ± 40	85.6	93.5	1.1	3.7
Carrier 6	-	-	-	-	-	-	-
Carrier 7	84.2	0.9 ± 0.4	104 ± 26	21.9	26.0	3.9	1.5
Carrier 12	73.5	7.1 ± 2.4	828 ± 397	69.3	94.2	10.3	1.5
Carrier 8	82.1	1.9 ± 0.3	315 ± 59	77.3	94.2	2.4	4.3
Carrier 9	82.1	1.1 ± 0.5	175 ± 3	76.5	93.2	1.5	4.3
Carrier 10	83.5	2.1 ± 0.3	186 ± 43	71.7	85.9	2.9	3.9
Carrier 11	83.0	1.0 ± 0.3	165 ± 104	80.8	97.4	1.2	4.5
Carrier 13	76.0	3.5 ± 0.6	929 ± 231	70.2	92.5	4.9	2.1
Carrier 14	80.6	1.6 ± 0.5	364 ± 156	73.3	90.9	2.2	2.4
Carrier 15	81.4	4.5 ± 1.0	234 ± 90	17.6	21.6	25.7	0.5
Carrier 16	81.6	4.4 ± 1.0	512 ± 197	64.2	78.6	6.8	2.4
Carrier 17	112.0	3.4 ± 1.1	565 ± 71	97.9	87.4	3.5	2.7
Carrier 18	111.5	3.0 ± 0.01	90 ± 55	37.4	33.5	7.9	1.3

**Figure 3:**
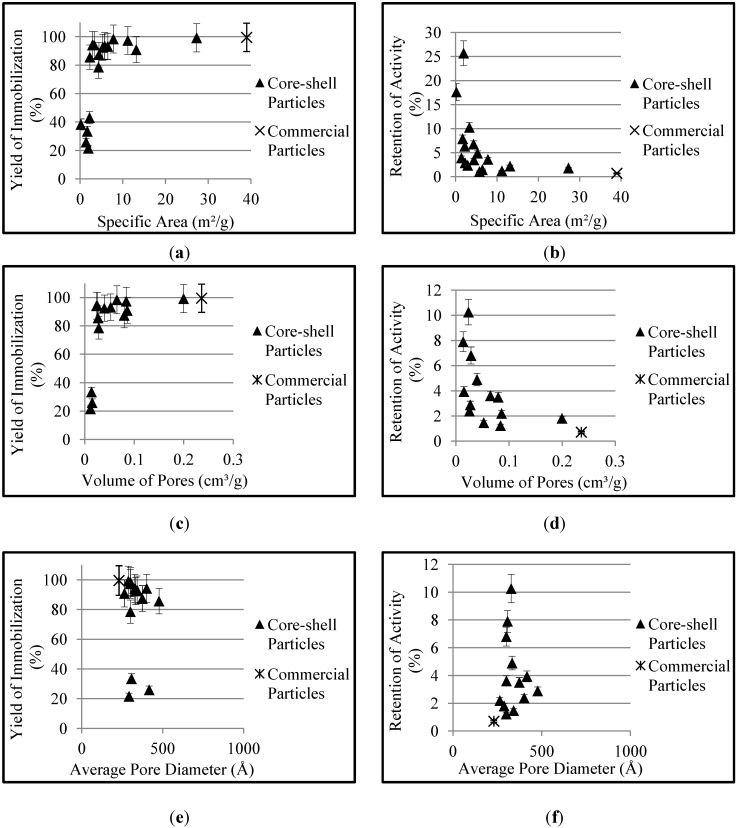
Effect of morphological properties of the supports on the immobilization parameters: (**a**) specific area *vs.* yield of immobilization; (**b**) specific area *vs.* retention of activity; (**c**) volume of pores *vs.* yield of immobilization; (**d**) volume of pores *vs.* retention of activity; (**e**) average pore diameter *vs.* yield of immobilization; (**f**) average pore diameter *vs.* retention of activity.

In order to facilitate the understanding of the relationship between the morphological properties of the supports and the immobilization parameters, some graphs were prepared as shown in Figure 3. It is possible to observe in Figure 3a that there exists a relatively low minimum specific area value that virtually guarantees the complete adsorption of the initial amount of protein, indicating the good interaction of the enzyme and the support surfaces. It can also be observed in Figure 3b that the increase of the specific area causes the reduction of the retention of activity, due to the accumulation of polymer in the shell, indicating the existence of diffusional limitation effects. Diffusional limitation is probably related to the high reaction rates, as reaction dynamics is very fast and steady state concentration profiles are not likely to be attained. The reduction of retention of activity can also be related to the formation of longer pores in the shell, as discussed in the literature [15], as the presence of long pores in the shell can lead to low accessibility of reagents present in the aqueous media due to the hydrophobicity of the support.

It can be noted in Figure 3c that there also is a minimum value of volume of pores required for almost complete adsorption of protein and that the retention of activity decreases with the increase of the volume of pores, as shown in Figure 3d. The effects caused by the specific area and the volume of pores on the immobilization parameters are similar because these two morphological properties respond primarily to the accumulation of polymer in the shell, so that they should be regarded as redundant dependent variables.

Figure 3e,f apparently indicate that the average pore diameter does not affect the yield of immobilization and retention of activity. According to Mojovic *et al.*, the characteristic diameter of lipases is close to 70 Å [37]. Table 1 and Figure 3e,f show that the average pore diameters of the supports were significantly larger than the characteristic size of the enzyme, which can probably explain the secondary effect of average pore diameters on the immobilization parameters. Although three samples apparently showed a distinct behavior, this can probably be associated with the low specific areas of these supports, not considered in Figure 3e,f. Bayne *et al.*, also noticed the lack of significant correlation between the pore diameters and the retention of activity and between the pore characteristics and the protein loading level in supports of different compositions, for large pore diameters, as observed in the present manuscript [38].

### 2.3. Influence of the Morphology of the Supports on the Hydrolytic Activities of the Biocatalysts

According to Table 1, it is possible to note that the absolute values of the hydrolytic activities of the biocatalysts were low. This could already be expected because CAL-B is employed mainly to catalyze esterification reactions. Surprisingly, particle cores with very low specific areas presented very high hydrolytic activities. This can be explained in terms of the high exposure of adsorbed enzyme molecules to the substrate, as adsorbed enzyme molecules are located essentially on the outer surface of the particle, which substantially minimizes possible diffusion effects. This result supports the idea that mass transfer limitations can control the performances of the produced biocatalysts for the hydrolytic reaction. It is also very important to notice that the majority of the produced biocatalysts exhibited higher hydrolytic activities than the biocatalyst synthesized with the commercial support, indicating that the performances of the produced supports can be regarded as good.

**Figure 4:**
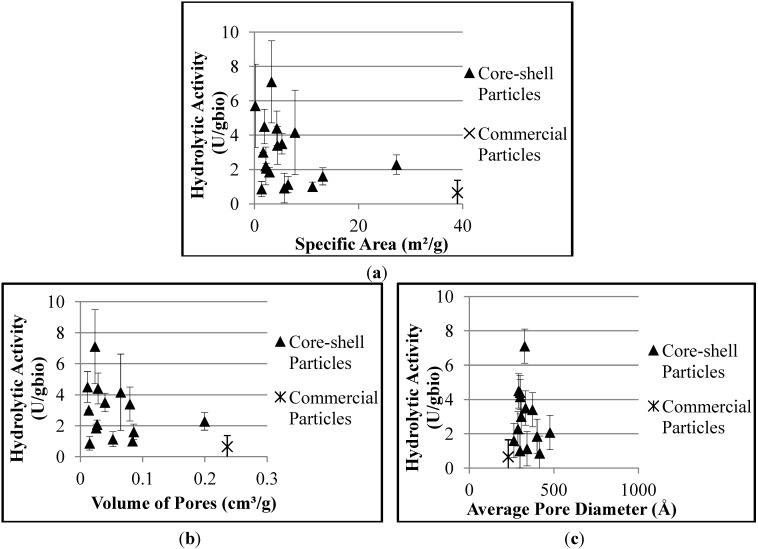
Effect of morphological properties of the supports on the hydrolytic activities of the produced biocatalysts: (**a**) Specific area; (**b**) Volume of pores; (**c**) Average pore diameter.

The effects of the morphological properties of the supports on the hydrolytic activity of the biocatalysts are shown in Figure 4. It is possible to observe the decrease of hydrolytic activity with the specific area and volume of pores, while the average pore diameter apparently does not affect the hydrolytic activity of the biocatalyst, for the reasons presented before.

It must be noted that the increase of the monomer feed flowrate caused the decrease of the hydrolytic activities of the biocatalysts, probably because of the decrease of the amount of enzyme adsorbed onto de supports (due to the lower specific areas) and the increase of the diffusional limitation effects (as the increase of monomer feed flowrate accelerates particle agglomeration on the shell). On the other hand, the increase of the amount of emulsifier caused the increase of the hydrolytic activity, while the increase of the amount of initiator caused the decrease of the hydrolytic activity of the biocatalyst. Both variables affect the rate of formation and the diameters of the emulsified particles in a complex manner, also affecting the hydrophobicity of the particle surfaces. Therefore, it is possible to note that the polymerization conditions and the emulsion recipe do affect the immobilization parameters and the final biocatalyst performance, as presented below with more detail.

### 2.4. Influence of the Morphology of the Supports on the Esterification Activities of the Biocatalysts

The esterification activities of the biocatalysts are also shown in Table 1. As mentioned previously, the absolute values of the esterification activities are much more significant than the hydrolytic activities. Particularly, core particles presented very low esterification activities, given the lower amounts of adsorbed enzyme. It is important to observe that diffusional effects are expected to be more intense in hydrolytic reactions, due to the higher rates of reaction. Esterification reactions are less intense and take place for longer periods of time, which probably can explain the differences observed in both cases. Once more, it is important to emphasize that many produced biocatalysts exhibited higher esterification activities than the biocatalyst synthesized with the commercial support, indicating that the performances of the produced supports can be regarded as good.

**Figure 5:**
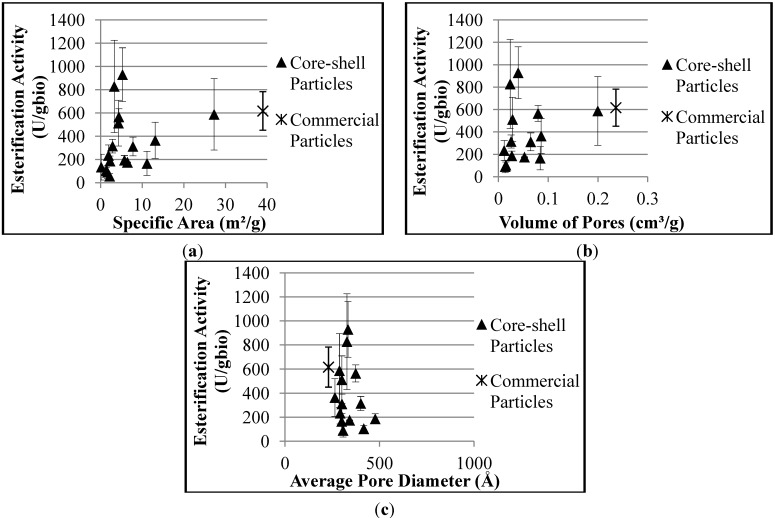
Effect of morphological properties of the supports on the esterification activities of the produced biocatalysts: (**a**) specific area; (**b**) volume of pores and (**c**) average pore diameter.

Figure 5 shows the effect of morphological properties of the supports on the esterification activities of the obtained biocatalysts. The increase of the specific area (and of the volume of pores) tends to increase of the esterification activities, due to the higher amounts of enzyme retained by the supports. Apparently, as observed previously for the hydrolytic activity, the average pore diameter does not affect the esterification activity of the biocatalyst.

The increase of the monomer feed flowrate caused the decrease of the esterification activities of the produced biocatalysts, as also observed previously for hydrolytic activities and reinforcing that diffusional limitations induced by thicker polymer shells can control the enzymatic activities of the biocatalysts. On the other hand, the increase of emulsifier amounts caused the increase of the esterification activity, while variation of the initiator amounts did not affect the esterification activity of the biocatalysts very significantly. As already said, both variables affect the rate of formation and the diameters of the emulsified particles in a complex manner, also affecting the hydrophobicity of the particle surfaces, which can certainly affect the final performances of the biocatalysts. It is also possible to notice that the increase of the amount monomer caused the increase of esterification activities of the biocatalysts. This can probably be explained in terms of the increase of protein retained on the supports with the increase of the amount of monomer, given the higher specific areas of the support. Once more, it is possible to note that the polymerization conditions and the emulsion recipe do affect the immobilization parameters and the final biocatalyst performance.

### 2.5. Empirical Models

Based on the previous results, empirical models were developed to describe observed hydrolytic and esterification activities of the biocatalysts as functions of the morphological properties of the supports. It is important to emphasize that empirical models built as functions of the operation variables of the polymerization process led to worse quantitative results, when compared to empirical models built as functions of the morphological properties of the support. This probably indicates that the morphological properties exert a much more significant effect on the final biocatalysts performances than the polymerization operation conditions, although morphological properties resulted from reaction operation conditions. For this reason, empirical models are presented only for enzymatic activities as functions of the morphological properties of the supports.

**Figure 6:**
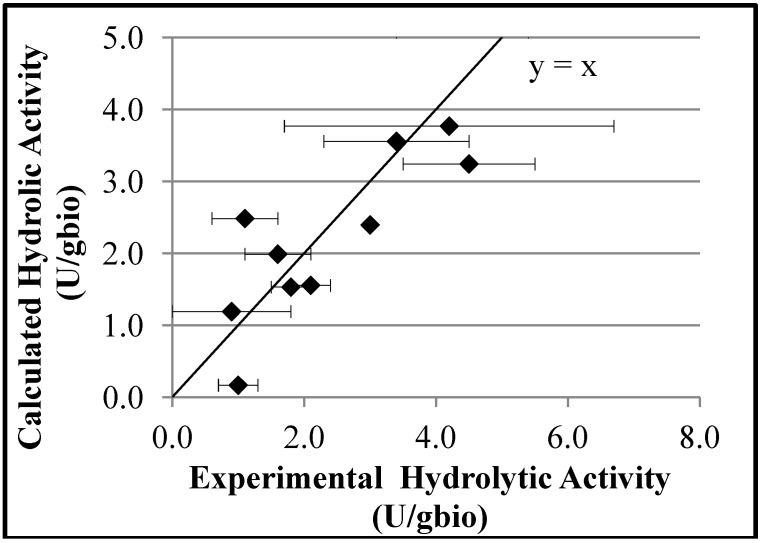
Relationship between the experimental hydrolytic activities and the hydrolytic activities predicted by the empirical model.

The empirical model that describes the hydrolytic activity is shown in Table 2. It can be observed that the performance of the hydrolytic model is satisfactory, with correlation coefficient of 0.84. Figure 6 illustrates the relationship between the experimental hydrolytic activities of the biocatalysts and the hydrolytic activity calculated by the model, showing that calculated errors are similar to observed experimental fluctuations. According to the empirical model, a maximum hydrolytic activity value is reached as the specific areas of the supports increase. This result corroborates the idea that diffusional limitation effects influence the performances of the biocatalysts, probably because of the high reaction rates, as already discussed. As shown before, average pore diameters exert secondary effects on the hydrolytic activities, through the nonlinear interaction effects (parameters b12 and b13). Although values of specific areas and volumes of pores are correlated, inclusion of both variables on the model was necessary, indicating that these variables respond independently to other process perturbations.

**Table 2:** Empirical model that describes the hydrolytic activity of the produced biocatalysts.

Equation					
A_hid_ = a1 × S_esp_ + a3 × V_esp_ + b11 × S_esp_ × S_esp_ + b12 × V_esp_ × D_p_ + b13 × S_esp_ × D_p_					
Estimated Parameters (R = 0.84; Degree of Freedom = 6)					
Parameters	a1 (U∙m^−2^)	a3 (10^6^∙U∙m^−3^)	b11 (U∙g·m^−4^)	b12 (10^16^∙U∙m^−4^)	b13 (10^10^∙U∙m^−3^)
Estimated Values	8.51	−532.97	−0.16	1.52	−0.02
Standard Errors	2.20	239.72	0.03	0.65	0.01
Significance	0.992	0.932	0.999	0.942	0.986

Notes: where A_hid_ is the hydrolytic activity of the immobilized enzymes (U/gbio); S_esp_ is the specific area of the supports (m^2^/g); D_p_ is the average pore diameter (Å); V_esp_ is the volume of pores of the supports (cm^3^/g).

**Figure 7:**
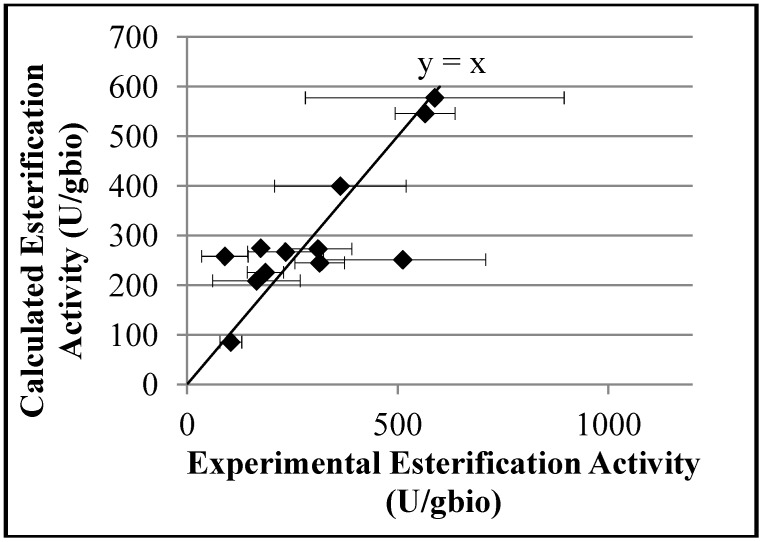
Relationship between the experimental esterification activities and the esterification activities predicted by the empirical model.

The empirical model developed to describe the esterification activities is shown in Table 3 and Figure 7. As a whole, the model performance can be regarded as satisfactory (R = 0.88), despite the larger number of parameters and the very poor significance of the calculated parameters. The poor significance of model parameters is explained by the very small number of degrees of freedom of the resulting model and the natural fluctuations of the available experimental data. On the other hand, removal of model parameters can lead to dramatic drop of the correlation coefficient. This probably indicates that additional factors also affect the esterification activities of final biocatalysts, although not considered in the proposed empirical model. For instance, the existence of diffusional limitation effects can also indicate that the enzyme molecule is subject to conformational modification in the different biocatalysts, which is not considered here. Besides, as emulsifier and initiator molecules can change the hydrophobicity of the emulsified polymer particles that form the shell, this effect should also be investigated in future works.

**Table 3:** Empirical model that describes the esterification activity of the produced biocatalysts.

Equation				
A_ester_ = a0 + a1 × S_esp_ + a2 × D_p_ + a3 × V_esp_ + b11 × S_esp_ × D_p_ + b12 × V_esp_ × S_esp_ + b13 × V_esp_ × D_p_ + c12 × S_esp_ × D_p_ × V_esp_				
Estimated Parameters (R = 0.88; Degree of Freedom = 3)				
Parameters		Estimated Values	Standard Errors	Significance
a0	(U·g^−1^)	−19.5	1204.1	0.012
a1	(U·m^−2^)	652.6	850.3	0.501
a2	(10^10^ U·(g·m)^−1^)	−0.2	3.6	0.032
a3	(10^6^ U·m^−3^)	−39898.7	58152.5	0.458
b11	(10^10^ U·m^−3^)	−1.7	2.4	0.426
b12	(10^6^ U·g·m^−5^)	−1977.8	7867.3	0.182
b13	(10^16^ U·m^−4^)	147.3	148.8	0.605
c12	(10^16^ U·g·m^−6^)	0.4	25.3	0.011

Notes: where A_ester_ is the esterification activity of the immobilized enzymes (U/g_bio_); S_esp_ is the specific area of the supports (m^2^/g); D_p_ is the average pore diameter (Å); V_esp_ is the volume of pores of the supports (cm^3^/g).

## 3. Experimental Section

### 3.1. Materials

All reactants were used as received, without any purification step. Lipozyme® CALB L, Lipase B from *Candida antarctica* (CAL-B) was supplied by Novozymes (Bagsvaerd, Denmark) in the soluble form. The substrate used for determination of hydrolytic activities, *p*-nitrophenyl laurate (*p*-NPL), was purchased from Sigma Aldrich (St. Louis, MO, USA) with minimum purity of 98 wt %. Ethanol P.A., supplied by Vetec Química Fina (Rio de Janeiro, Brazil), with a minimum purity of 99.8 wt %, was used for washing of the polymer supports and also as substrate in esterification reactions. Oleic acid with minimum purity of 98 wt % was supplied by Vetec Química Fina and was used as substrate in esterification reactions. The commercial polypropylene support Accurel^®^ MP 1000 was supplied by Akzo Nobel Faser AG (Obernburg, Germany) and used as a benchmark support for comparative evaluation of the performances of the synthesized biocatalysts. The Bradford reagent was supplied by BioRad (Hercules, CA, USA). Styrene with minimum purity of 99.5 wt % used as monomer in polymerization reactions was supplied by Sigma Aldrich (St. Louis, MO, USA). Other reagents and solvents used for product purification and characterization were of analytical grade.

### 3.2. Preparation of Core-Shell Polymer Supports

Core-shell polystyrene supports were produced through simultaneous suspension/emulsion polymerizations. The analyzed polymerization technique comprises two fundamental steps. Initially, the particle cores are formed through standard batch suspension polymerization. After the specified amount of time, the emulsion constituents are added to the reacting medium, forming small particles through emulsion polymerization. The newly formed particles coagulate over the cores produced previously, forming the shell and leading to formation of porous core-shell polymer particles [28,29,39]. It is important to note that the constituents and reaction conditions used in the emulsion polymerization step affect the final morphology of the produced particles [28,29,39].

Combined suspension-emulsion polymerizations were carried out as described in the literature [28,29,39]. Reactions were carried out in an open 1-L jacketed glass reactor (FGG Equipamentos Científicos, São Paulo, Brazil) equipped with a thermostatic bath (Haake Phoenix II model, Thermo Scientific, Karlsruhe, Germany), used to control the reactor temperature (kept at 85 °C in all experiments), and with a cooling bath (Nova Ética, São Paulo, Brazil), used to control the temperature of the condenser (kept at 10 °C in all experiments). Styrene was used as monomer in all reactions. Initially, the particle cores were formed through standard batch suspension polymerization by dispersing 100 g of an organic solution (containing styrene and 3.8 wt % of the initiator benzoyl peroxide) in 370 g of an aqueous solution (containing distilled water and 0.80 wt % of poly(vinyl alcohol), used as stabilizer) through agitation (kept equal to 950 rpm in all runs). After two hours of suspension reaction, the emulsion constituents (styrene monomer and an aqueous solution containing distilled water, 0.13 wt % of the initiator potassium persulfate and 1 wt % of lauryl sulfate, used as emulsifier) were added to the reacting medium. In order to control the reaction temperature, part of the emulsion feed (30 g of monomer and 230 g of the aqueous solution) was added initially in a single load, while the remaining 70 g of monomer were added continuously at the specified flow rate. The experimental run was finished after 6 hours of reaction. Supports with distinct properties were synthesized through manipulation of monomer feed flow rates (V), amounts of emulsifier, amounts of initiator and amounts of monomer that were used in the emulsion polymerization.

At the end of the batch, the reactor was cooled down and the obtained particles were filtrated and washed with abundant cold water. Finally, the obtained polymer particles were dried in a vacuum oven at ambient temperature until constant mass. The analyzed experimental design is presented in Table 4. Experiments did not follow a standard factorial design because combination of certain process operation conditions led to massive coagulation of the polymer particles.

**Table 4:** Reaction conditions and morphological properties of the produced supports: specific area (S_esp_), volume of pores (V_esp_) and average pore diameter (D_p_). Variations regard the emulsion polymerization step and used recipe defined in Section 3.2 as reference.

Reaction	Legend	Graph Legend	Sesp (m^2^/g)	Vesp (cm^3^/g)	Dp (Å)
1 ^1^	Floating Flow Rate	Floating Flow Rate	27.3	0.20	287.6
2 ^2^	Core	Core	0.2	-	-
3	Reference; V = 0.035 L/h	V = 0.035 L/h	7.8	0.06	300.9
4 ^3^	Reference; V = 0.039 L/h	V = 0.039 L/h	2.2	-	-
5 ^3^	Reference; V = 0.071 L/h	V = 0.071 L/h	5.7	-	-
6 ^4^	Reference; V = 0.127 L/h	V = 0.127 L/h	-	-	-
7 ^3^	Reference; V = 0.082 L/h	V = 0.082 L/h	1.4	0.01	416.4
8	+25% Emulsifier; V = 0.035 L/h	+25% Emulsifier	2.9	0.03	400.8
9	−25% Emulsifier; V = 0.035 L/h	−25% Emulsifier	6.5	0.05	341.4
10	−25% Initiator; V = 0.035 L/h	−25% Initiator	2.3	0.03	477.0
11	+25% Initiator; V = 0.035 L/h	+25% Initiator	11.2	0.08	299.7
12	Reference; V = 0.017 L/h	V = 0.017 L/h	3.3	0.02	327.6
13	−25% Initiator; − 50% Monomer; V = 0.035 L/h	−Initiator; −Monomer	5.3	0.04	333.0
14	−25% Initiator; + 50% Monomer; V = 0.035 L/h	−Initiator; +Monomer	13.1	0.09	263.7
15	−50% Monomer; V = 0.035 L/h	0 Initiator; −Monomer	1.9	0.01	291.8
16	+50% Monomer; V = 0.035 L/h	0 Initiator; +Monomer	4.3	0.03	300.4
17	Reference; V = 0.035 L/h	0 Initiator; 0 Monomer	4.4	0.08	373.7
18	−25% Initiator; V = 0.035 L/h	−Initiator; 0 Monomer	1.7	0.01	306.9
-	Commercial Support	Accurel	39	0.24	230.0

Notes: ^1^: Monomer feed rate was allowed to fluctuate around reference; ^2^: A shell was not formed; ^3^: Experiments performed for analysis of the maximum allowed monomer feed rate; ^4^: Massive agglomeration of particles, characterizing a maximum limit for monomer feed rate.

### 3.3. Characterization of Supports Morphology

**Figure 8:**
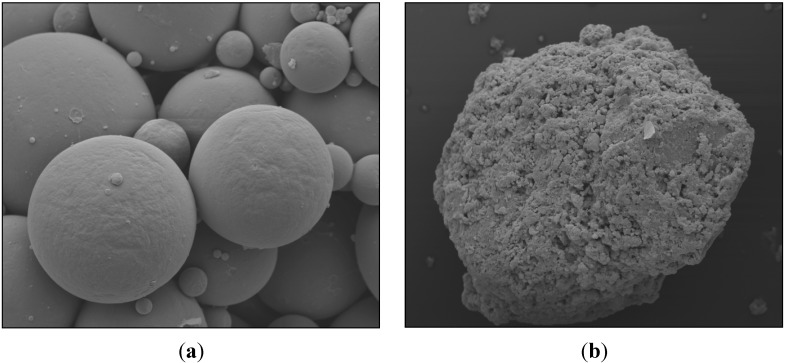
Morphological aspect of the produced supports: (**a**) cores and (**b**) core-shell particles.

In order to determine the specific area, the average pore diameter and the volume of pores of the produced core-shell particles, a standard BET surface analyzer was used (ASAP 2020 model, supplied by Micromeritics, Norcross, GA, USA). This rather standard characterization technique is based on the adsorption of nitrogen on the surface of the particles, as described in details elsewhere [2,28,29,30]. Prior to analyses, samples were pretreated under vacuum at 60 °C. Properties of the obtained supports (specific area, volume of pores and average pore diameter) are also shown in Table 4. Figure 8 illustrates the morphological aspect of the produced particles. The commercial support, Accurel^®^ MP1000, was also characterized at similar conditions in order to provide reference values for evaluation of the performance of the obtained core-shell particles. As observed experimentally, the analyzed Accurel^®^ MP1000 samples presented specific area of 39 m^2^/g, average pore diameter of 230 Å and volume of pores of 0.236 cm^3^/g.

### 3.4. Immobilization Procedure

The immobilization procedure used here and definition of the immobilization parameters are described in detail elsewhere [2,40,41,42]. Firstly, the supports were washed in order to eliminate possible residual monomer and to facilitate the penetration of the aqueous enzyme into the porous particles during the enzymatic immobilization. Initially, 10 mL of ethanol were added to 1 g of dry support. After 30 min, particles were filtrated and 10 mL of distilled water were added. After 10 min, particles were filtrated again and washed with abundant distilled water. Finally, the supports were washed with sodium phosphate buffer (5 mM, pH = 7.0) and kept in the refrigerator (17 °C) until immobilization.

Cunha observed pronounced decrease of the retention of hydrolytic activity when the hydrolytic activity of the enzyme solution was increased from 100 U to 200 U in presence of 1 g of support, using *p*-NPL as substrate (activities are described in the next sections) [40]. For this reason, the initial hydrolytic activity of the enzymatic solution was kept equal to 100 U in all immobilization experiments and in presence of 1 g of support. In order to do that, the commercial enzyme solution was dissolved in sodium phosphate buffer (5 mM) in order to obtain the desired enzymatic activity of 100 U. The immobilization process was initiated by adding 10 mL of the enzymatic solution with total hydrolytic activity of 100 U to 1 g of treated and washed support. The immobilization proceeded for 26 h under mild stirring at 4 °C. During immobilization, aliquots of 150 µL were collected (0, 30 min, 1 h, 3 h, 6 h, 26 h) for evaluation of the hydrolytic activity of the aqueous phase, using *p*-NPL as substrate [2,40,41,42]. Finally, the obtained biocatalyst was dried in desiccators for one week and stored in a refrigerator (17 °C). It is important to mention that some measurements were performed in triplicate and that the experimental errors of hydrolytic activities were calculated with a confidence level of 95%.

### 3.5. Hydrolytic Activity of Biocatalysts

The biocatalysts were characterized in terms of the hydrolytic activity. Hydrolysis reactions were carried out in batch mode. Initially, 9 mL of sodium phosphate buffer (25 mM, pH = 7.0) and 1 mL of the substrate solution were added to a reactor. Then, approximately 10 mg of the biocatalyst were added to the reactor, initiating the hydrolysis reaction. Each reaction was conducted under mild stirring at 30 °C. Aliquots of 700 µL were collected during the batch for approximately 6 min. The absorbance of each aliquot was analyzed in a spectrophotometer (UV-1800 model, supplied by Shimadzu, Kyoto, Japan) at 30 °C at the wavelength of 412 nm. It is important to mention that experiments were analyzed in triplicates and that the experimental errors of hydrolytic activities were calculated with confidence level of 95%. It is important to note that one unit of enzymatic activity (1 IU or 1 U) corresponds to the amount of enzyme necessary to produce 1 μmol of *p*-nitrophenol per minute under the described operating conditions. It is also important to mention that the expression U/g_support_ is related to the enzymatic activity associated to 1 g of polymer support and that the expression U/g_bio_ corresponds to the enzymatic activity of 1 g of biocatalyst, after immobilization of the enzyme.

### 3.6. Immobilization Parameters

The first analyzed immobilization parameter, the enzyme yield of immobilization, was determined as shown in Equation (1):

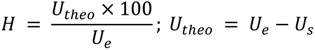
(1)
where *H* is the enzyme yield of immobilization (%); *U_theo_* is the total immobilized enzyme activity in terms of the amount of support (U/g_support_); *U_e_* is the total enzyme activity of the solution in the beginning of the immobilization process (U/g_support_) and *U_s_* is the total enzyme activity of the solution at the end of the immobilization process (U/g_support_). A second analyzed immobilization parameter, the enzyme retention of activity, was determined as shown in Equation (2):

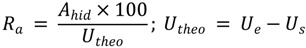
(2)
where *R_a_* is the enzyme retention of activity (%) and *A_hid_* is the hydrolytic activity of the biocatalyst (U/gbio).

### 3.7. Esterification Activity of Biocatalysts

The esterification activities of the biocatalysts were also evaluated. Esterification reactions were carried out in a jacketed reactor at 40 °C under mild stirring. Oleic acid (10.1 mL) and ethanol (1.9 mL) were used as substrates, using 0.2 g of biocatalyst in the reaction medium. Aliquots of 100 μL were collected at different times for evaluation of the activities (0, 10 min, 20 min, 30 min, 40 min, 60 min). Aliquots were added to a mixture of ethanol and acetone (1:1) (v/v) and the oleic acid content was determined by titration with NaOH (0.02 M), using an automatic titrator (G20 model, Mettler Toledo, Schwerzenbach, Switzerland). It is important to mention that experiments were analyzed in triplicates and that the experimental errors of esterification activities were calculated with confidence level of 95%. It is important to note that one unit of enzymatic activity (1 IU or 1 U) corresponds to the amount of enzyme necessary to form 1 μmol of ethyl oleate per minute under the described operating conditions. The expression U/g_bio_ corresponds to the enzymatic activity of 1 g of biocatalyst, after immobilization of the enzyme. The enzyme activity (*A_ester_*) was determined only in the linear region of the product concentration trajectory.

### 3.8. Determination of Protein Concentration

Protein concentrations in the supernatant during the immobilization process were evaluated in order to confirm the results obtained through the enzymatic activity analyses. Analyses were performed with help of the well-known Bradford method. Calibration was performed with bovine serum albumin (BSA) and light absorbance of the Bradford reagent at the wavelength of 595 nm [43], using a spectrophotometer (Power Wave XS model, BioTek, Winooski, VT, USA).

It is important to mention that some measurements were performed in triplicates and that the experimental errors of protein concentrations were calculated with confidence level of 95%.

### 3.9. Statistical Analysis

Statistical analyses and empirical model building were performed with help of the Statistica Software, version 7.0, developed by Stat Soft Inc. (Tulsa, OK, USA). The modeling step was performed iteratively. Models were proposed and the correlation coefficients between the available experimental data and model calculations were computed. Models were discarded when correlation coefficients were smaller than 0.7. When correlation coefficients were sufficiently close to 1 (for instance, above 0.8), the significance of model parameters was calculated. The main objective of the iterative procedure was the obtainment of models that could satisfactorily describe the available data with the smallest possible number of parameters.

## 4. Conclusions

Polystyrene core-shell particles were produced through combined suspension/emulsion polymerizations under different conditions, leading to production of polymer supports with distinct morphological properties (specific areas, volume of pores and average pore size). These supports were used for immobilization of lipase B from *Candida antarctica* (CAL-B), allowing for production of different biocatalysts. The performances of the biocatalysts were characterized in terms of hydrolytic and esterification activities. The obtained results indicated that modification of the reaction operation conditions can lead to significant changes of the morphological properties of the supports, leading to biocatalysts that present very different final performances. Particularly, the performances of some biocatalysts prepared at different conditions were better than the ones prepared with commercial supports, indicating that the combined suspension/emulsion polymerization technique can provide an interesting route for production of enzyme supports. It was observed that the specific area of the supports was the most influential morphological parameter in the analyzed system, although the increase of the specific area caused the decrease of the retention of activity, due to diffusional limitation effects. The specific area and volume of pores of the obtained supports were correlated to each other, as both variables depend on the accumulation of polymer material on the particle shell. The average pore diameter of the support did not affect the performances of the biocatalysts very significantly, probably because of the small diameter of CAL-B and the relatively large pore sizes of the supports.
